# National trends in hydrocephalus mortality in the United States, 1999–2024: a serial cross-sectional (ecological) trend analysis

**DOI:** 10.3389/fpubh.2026.1896078

**Published:** 2026-08-05

**Authors:** Zhongbo Yang, Chenxi He, Xiang Wu, Jinning Song

**Affiliations:** 1 Department of Neurosurgery, The First Affiliated Hospital of Xi‘an Jiaotong University, Xi'an, China; 2 Xi‘an Children's Hospital, Xi'an, China; 3 Department of Neurology, Shaanxi Provincial People's Hospital, Xi'an, Shaanxi, China

**Keywords:** age-adjusted mortality rates, annual percent change, average annual percent change, hydrocephalus mortality, joinpoint regression

## Abstract

**Background:**

How did hydrocephalus-attributable mortality trends and disparities evolve in the United States from 1999 to 2024, and what role did the COVID-19 pandemic play in shaping these patterns during and following its onset?

**Methods:**

Using national mortality data from the CDC WONDER database (1999–2024), we assessed age-adjusted mortality rates (AAMRs) for hydrocephalus among population aged 0–85+ years. Temporal trends were evaluated via joinpoint regression to calculate annual percent change (APC) and average annual percent change (AAPC) with 95% confidence intervals, stratified by age, gender, ethnicity, census region, and state level.

**Results:**

From 1999 to 2024, a total of 20142 hydrocephalus-related deaths were identified and the overall AAMR for the U.S. population showed a slow but no significant change (AAPC −0.35%, 95% CI: −0.76 to 0.06, *P* = 0.094), initially decreasing until 2019 before reversing to an increasing trend thereafter. Males had consistently higher mortality than females, with both sexes exhibiting an initial decline followed by a later increase. By age group, the youngest cohort (0–24 years) experienced the steepest decline, while the middle-aged group (25–54 years) showed a modest increase, and the oldest group (≥55 years) displayed a slight decline. Both ethnic groups exhibited a pattern of early decline followed by recent increase, with non-White populations showing steeper changes in both directions. Geographically, both the Northeast and West regions showed an early decline followed by a recent increase, with the Northeast experiencing the largest rise (2014–2024: APC = 5.83, *P* < 0.001). Oklahoma and West Virginia displayed the steepest percentage increases nationwide (25.4%−62.5%), though these changes did not reach statistical significance. When comparing pre-pandemic (1999–2019) and pandemic (2020–2023) periods, mortality trends reversed from a significant decline (AAPC = −0.87, *P* = 0.003) to a non-significant increase (AAPC = 3.61, *P* = 0.12), with notable increases among older adults, non-White populations, and residents of the Northeast and West regions.

**Conclusions:**

In summary, while the overall 26-year trend was stable, this stability masked a consistent reversal pattern across most demographic and geographic subgroups—an early decline followed by a recent increase—most notably among older adults, non-White populations, and residents of the Northeast and West regions. These findings highlight the need for continued surveillance and further investigation into the drivers of this recent shift, while also pointing to the potential value of maintaining equitable access to specialized neurosurgical care for vulnerable populations.

## 1 Introduction

Hydrocephalus is a common and complex neurological disorder characterized by pathological alterations in cerebrospinal fluid (CSF) homeostasis, resulting in active distension of the cerebral ventricular system (1). The condition arises from an imbalance between CSF production and absorption, most frequently due to impaired CSF flow or reduced absorption capacity rather than CSF overproduction (2, 3). The choroid plexus, a richly vascularized structure, secretes roughly 500 mL of CSF daily in adults, while the total CSF volume remains stable at about 150 mL due to precisely controlled flow and reabsorption (4, 5). Hydrocephalus, one of the most common pathologies treated by neurosurgeons, which can exist as a disease itself, but is more often caused by other diseases. Congenital or acquired impediments can disrupt the CSF circulation and result in hydrocephalus (6). Myelomeningocele is one of the common congenital causes. Acquired disorders commonly include hemorrhages, central nervous system infections, trauma, tumors (1, 2, 7, 8). Hydrocephalus can be categorized into different types with distinct pathophysiologies: communicating hydrocephalus, obstructive hydrocephalus, normal-pressure hydrocephalus and other and unspecified hydrocephalus (9). The typical clinical manifestations of hydrocephalus include headache, nausea, vomiting, urinary incontinence, abnormal gait, etc. It is estimated that the incidence of hydrocephalus in children is about 400,000 new cases per year in the world (10), and a separate study suggests that the average global prevalence of hydrocephalus is 85 cases per 100,000 individuals (11). Besides, the highest incidence rates of hydrocephalus are observed in Africa and Latin America, with 145 and 316 cases per 100,000 births, respectively, while the lowest rate is found in the United States and Canada (68 per 100,000 births) (10). The chronic nature of hydrocephalus places a heavy burden on both the individual and society (10). The economic burden of hydrocephalus treatment has risen substantially in the U.S.A, with total national costs increasing from $332 million in 2009 to $418 million in 2013 (12). Further, the study has reported rising trends in hydrocephalus-related healthcare utilization and costs, particularly among pediatric and older adults populations, which may be attributable to improved survival rates, increased awareness, and evolving treatment paradigms. Moreover, disparities in hydrocephalus outcomes have been observed across different demographic groups, with variations in access to care, shunt failure rates, and long-term prognosis (12). A recent study reported that mortality in pediatric hydrocephalus did not differ significantly according to demographic factors; however, non-white and Hispanic individuals exhibited a higher proportion of deaths (13).

The COVID-19 pandemic has further exacerbated trends in hydrocephalus-related morbidity and mortality. Pandemic-related stressors, such as economic insecurity, social isolation, and disruptions to healthcare access, have led to delayed presentations and interruptions in neurosurgical follow-up for patients with underlying cerebrospinal fluid disorders. Although hydrocephalus is not traditionally recognized as a common neurological complication of SARS-CoV-2 infection, accumulating case-based evidence has suggested a potential pathogenic link between COVID-19 and the development or decompensation of hydrocephalus (14–16). While massive subarachnoid hemorrhage is well established as a predisposing factor for hydrocephalus, emerging observations indicate that COVID-19 may serve as a precipitating event in susceptible individuals with pre-existing alterations in cerebrospinal fluid dynamics (17). The temporal association between SARS-CoV-2 infection and the onset of acute hydrocephalus, coupled with radiographic progression in the absence of interval neurological change, supports the plausibility of this relationship (17). Nevertheless, reports of COVID-19-associated hydrocephalus remain rare, and the pathophysiological mechanisms—whether mediated by direct viral invasion, inflammatory cytokine storm, or disruption of the glymphatic system—require further investigation.

Although rising trends in hydrocephalus-related morbidity and costs have been documented, comprehensive analysis of outcomes during the COVID-19 pandemic remains insufficient. However, no significant evidence has demonstrated increased hydrocephalus mortality during the pandemic period. Nevertheless, the lack of adequate analyses regarding the pandemic's impact on hydrocephalus care underscores the need for further investigation to guide public health policy and identify populations requiring targeted interventions. The aim of this study was to present comprehensive trends in hydrocephalus-related mortality in the United States from 1999 to 2024, stratified by key demographic characteristics, including age, sex, ethnicity, and geographic region. Additionally, we sought to examine the impact of the COVID-19 pandemic on these mortality trends, with particular attention to changes observed during and shortly after the pandemic period (2020–2024). By providing updated population-level estimates, this study seeks to inform public health surveillance and highlight high-risk subgroups for targeted interventions.

## 2 Methods

### 2.1 Data sources and study variables

This cross-sectional study analyzed data from the CDC WONDER mortality database (1999–2024). Underlying cause-of-death files, derived from death certificates across all 50 US states and the District of Columbia, were extracted. Hydrocephalus-related mortality was identified using ICD-10 codes, including G91.0, G91.1, G91.2, G91.3, G91.8, and G91.9 (detailed in Table 1). The study adhered to STROBE reporting guidelines and was exempt from institutional review board review and informed consent due to the use of publicly available data (45 CFR §46) (18).

**Table 1:** Classification of diseases and codes (ICD-10) for hydrocephalus.

ICD-10 Code	Name
**G91**	Hydrocephalus
**G91.0**	Communicating hydrocephalus
**G91.1**	Obstructive hydrocephalus
**G91.2**	(Idiopathic) normal pressure hydrocephalus
**G91.3**	Post-traumatic hydrocephalus, unspecified
**G91.8**	Other hydrocephalus
**G91.9**	Hydrocephalus, unspecified

We examined deaths among persons aged 0–85+ years spanning 1999–2024. Age-adjusted annual mortality rates (per 100,000 population) were derived, with stratification by sex (male/female), age intervals (0–24, 25–54, and 55+ years), census regions (Northeast, Midwest, South and West), state, and ethnicity (non-White versus White) as classified by CDC WONDER from death certificates adhering to Office of Management and Budget standards. Using the National Center for Health Statistics approach, we age-standardized mortality rates to the 2000 US standard population. This process applies our study's age-specific rates to the 11 age categories of the 2,000 standard population, yielding weighted averages that facilitate meaningful comparisons of mortality trends across time. Of note, for subgroups with fewer than 20 deaths, CDC WONDER automatically suppresses the rates with an 'unreliable' flag; these estimates were excluded from the state-level analyses due to statistical instability. Consequently, state-level percentage changes should be interpreted with caution, as they are based on relatively small case numbers in certain strata. Besides, the data are therefore the final mortality data for 2024, as also reflected in NCHS's official 'Mortality in the United States, 204′ report released on January 29, 2026.

Graphical representations illustrated overall annual mortality rates for hydrocephalus, stratified according to sex, ethnicity, age, and census region. To determine whether the observed temporal trends reached statistical significance, joinpoint regression analyses were conducted (19, 20). The AAPC and APC along with 95% confidence intervals was calculated for each time segment identified by the joinpoint regression model.

### 2.2 Statistical analysis

All statistical analyses were carried out using Joinpoint Regression Program (version 6.0.1; Surveillance Research Program, National Cancer Institute). The Joinpoint program applies a regression model to time-series data to detect points where trends exhibit statistically significant changes. A two-tailed *P* value below.05 was considered statistically significant. All trend graphs were generated with R version 4.4.1 (36).

## 3 Results

### 3.1 Overall trends

**Figure 1:**
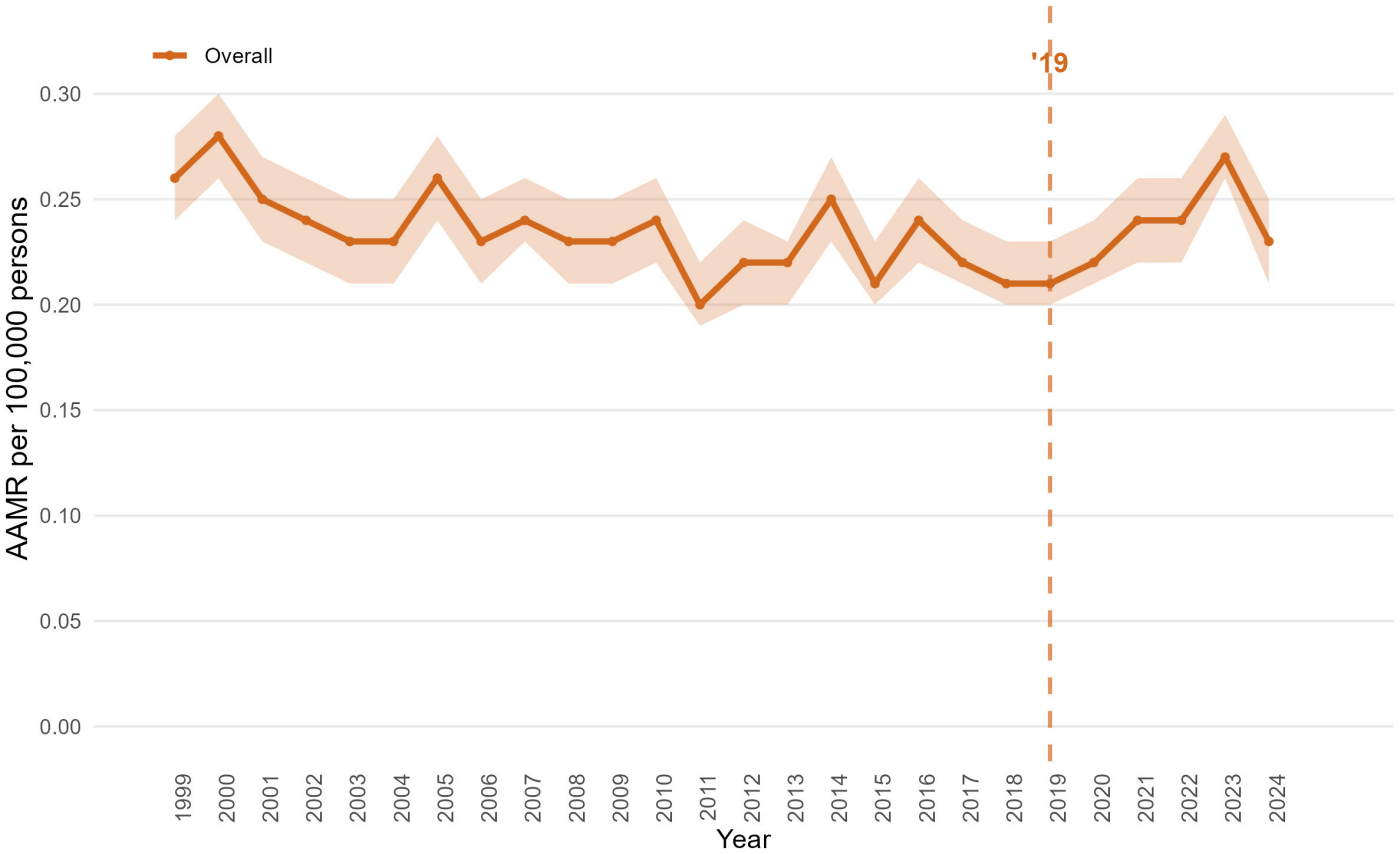
Overall trends in age-adjusted mortality rates for hydrocephalus, 1999–2024 (*N* = 20142). AAMR: age-adjusted mortality rates.

From 1999 to 2024, a total of 20142 hydrocephalus-related deaths were identified and the AAMR for the overall U.S. population decreased from 0.26 (95% CI: 0.24 – 0.28) in 1999 to 0.23 (95% CI: 0.21 – 0.25) in 2024, corresponding to an AAPC of 0.01 (−0.93 to 0.96, *P* = 0.98). Joinpoint regression analysis revealed that during 1999–2019, the AAMR showed a slow but statistically significant decline, with an APC of −0.87 (95% CI: −1.40 to −0.33, *P* = 0.003). Subsequently, during 2019–2024, the AAMR turned to an increasing trend, with an APC of 3.61 (95% CI: −0.97 −8.41, *P* = 0.12), although this rise did not reach statistical significance. A visual summary of these changes is available in Figure 1, with comprehensive data in Tables 2, 3.

**Table 2:** Trends in mortality from hydrocephalus in the United States, 1999–2024.

Characteristic	Measure (*N*)	Deaths_1999	Deaths_2024	AAMR_1999	AAMR_2024	AAPC (95% CI)
0	Overall (20142)	692	995	0.26 (0.24 to 0.28)	0.23 (0.21 to 0.25)	0.01 (−0.93 to 0.96)
1	Female (9254)	327	441	0.21 (0.19 to 0.23)	0.18 (0.16 to 0.20)	−0.46 (−1.47 to 0.56)
2	Male (10888)	365	554	0.34 (0.30 to 0.37)	0.29 (0.27 to 0.32)	−0.57 (−1.40 to 0.27)
3	0–24 years (1472)	100	53	0.12 (0.10 to 0.14)	0.03 (0.02 to 0.05)	−2.88 (−8.96 to 3.59)
4	25–54 years (2532)	67	120	0.07 (0.05 to 0.09)	0.10 (0.08 to 0.12)	1.42 (−0.91 to 3.80)
5	55+ years (15646)	525	822	0.90 (0.82 to 0.97)	0.82 (0.76 to 0.88)	−0.15 (−0.81 to 0.51)
6	non-White (3110)	107	184	0.26 (0.21 to 0.31)	0.24 (0.20 to 0.27)	−0.40 (−1.48 to 0.70)
7	White (17032)	585	811	0.25 (0.23 to 0.27)	0.22 (0.20 to 0.23)	−0.16 (−0.97 to 0.66)
8	Midwest (3133)	180	220	0.25 (0.22 to 0.29)	0.24 (0.21 to 0.28)	−0.16 (−1.35 to 1.04)
9	Northeast (4794)	102	174	0.18 (0.14 to 0.21)	0.23 (0.19 to 0.26)	0.57 (−0.67 to 1.82)
10	South (8287)	277	402	0.29 (0.26 to 0.33)	0.24 (0.21 to 0.26)	−0.38 (−1.16 to 0.40)
11	West (3952)	133	204	0.25 (0.20 to 0.29)	0.20 (0.17 to 0.23)	−0.58 (−1.49 to 0.34)

AAMR, age-adjusted mortality rates; AAPC, average annual percent change. Age-adjusted mortality rates by census region were calculated based on the deaths assigned to each region by CDC WONDER. The sum of regional death counts may slightly differ from the national total, as the national total is typically limited to the 50 U.S. states and the District of Columbia, while regional data may include deaths among U.S. residents from other territories or those with unspecified geographic information.

**Table 3:** Segmented trend analysis of average annual percent change (APC) for hydrocephalus in the United States from 1999 to 2024.

Characteristic	Segment 1			Segment 2		
	Year	APC (95% CI)	*P*-Value	Year	APC (95% CI)	*P*-Value
Overall	1999–2019	−0.87 (−1.40, −0.33)	0.003	2019–2024	3.61(−0.97, 8.41)	0.12
Sex						
Female	1999–2009	−3.52 (−5.46, −1.54)	0.001	2009–2024	1.64(0.43, 2.86)	0.01
Male	1999–2012	−2.13 (−3.50, −0.75)	0.004	2012–2024	1.15(0.08, 2.24)	0.04
Age						
0–24 years	1999–2019	−4.03 (−6.84, −1.13)	0.009	2019–2024	1.81 (−26.16, 40.36)	0.91
25–54 years	1999–2020	0.99 (−0.31, 2.31)	0.13	2020–2024	3.70 (−9.69, 19.07)	0.59
55+ years	1999–2012	−1.57 (−2.55, −0.58)	0.004	2012–2024	1.41 (0.42, 2.41)	0.007
Race						
Non-white	1999–2012	−2.68 (−4.31, −1.02)	0.003	2012–2024	2.14 (0.55, 3.75)	0.01
White	1999–2011	−1.77 (−3.05, −0.48)	0.009	2011–2024	1.35 (0.21, 2.52)	0.02
Census regions						
Midwest	1999–2015	−0.99 (−2.14, 0.18)	0.092	2015–2024	1.33 (−1.52, 4.27)	0.347
Northeast	1999–2014	−2.79 (−4.17, −1.40)	<0.001	2014–2024	5.83 (3.24, 8.49)	<0.001
South	1999–2011	−1.26 (−2.62, 0.12)	0.071	2011–2024	0.43 (−0.51, 1.39)	0.354
West	1999–2015	−2.55 (−3.56, −1.52)	<0.001	2015–2024	3.01 (1.00, 5.07)	0.005

APC, annual percent change; 95% CI, 95% confidence interval.

### 3.2 Trend by sex

**Figure 2:**
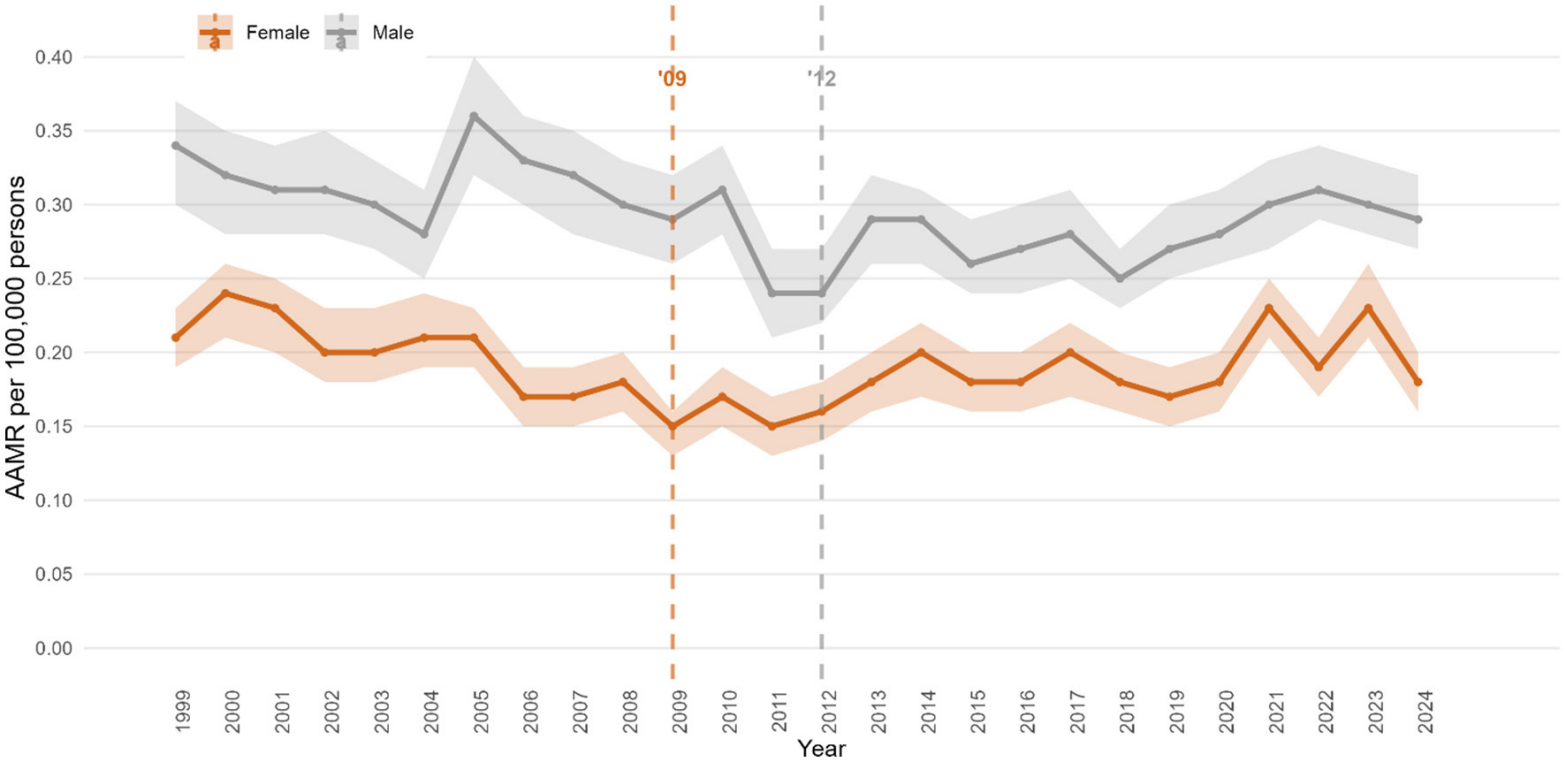
Sex-stratified trends in age-adjusted mortality rates for hydrocephalus, 1999–2024 (*N* = 20,142; females: 9,254; males: 10,888). AAMR: age-adjusted mortality rates.

Between 1999 and 2024, the AAMR among males fell from 0.34 (95% CI: 0.30 −0.37) to 0.29 (95% CI: 0.27 – 0.32), corresponding to an AAPC of −0.57 (95% CI: −1.40 – 0.27, *P* = 0.184). Temporal patterns for males exhibited two distinct segments: 1999–2012, with an APC of −2.13 (95% CI: −3.50 to −0.75, *P* = 0.004), followed by 2012–2024, where the APC was 1.15 (95% CI: 0.08 – 2.24, *P* = 0.04). For female individuals, the AAMR likewise dropped across the study interval, from 0.21 (95% CI: 0.19 – 0.23) in 1999 to 0.18 (95% CI: 0.16 – 0.20) in 2024, yielding an AAPC of −0.46 (95% CI: −1.47 – 0.56, *P* = 0.378). The female trend also changed over time: a decline during 1999–2009 with an APC of −3.52 (95% CI: −5.46 to −1.54, *P* = 0.001) and a subsequent rise from 2009 to 2024 showing an APC of 1.64 (95% CI: 0.43 – 2.86, *P* = 0.01). These results are displayed in Figure 2 and comprehensively reported in Tables 2, 3.

### 3.3 Trends by age

**Figure 3:**
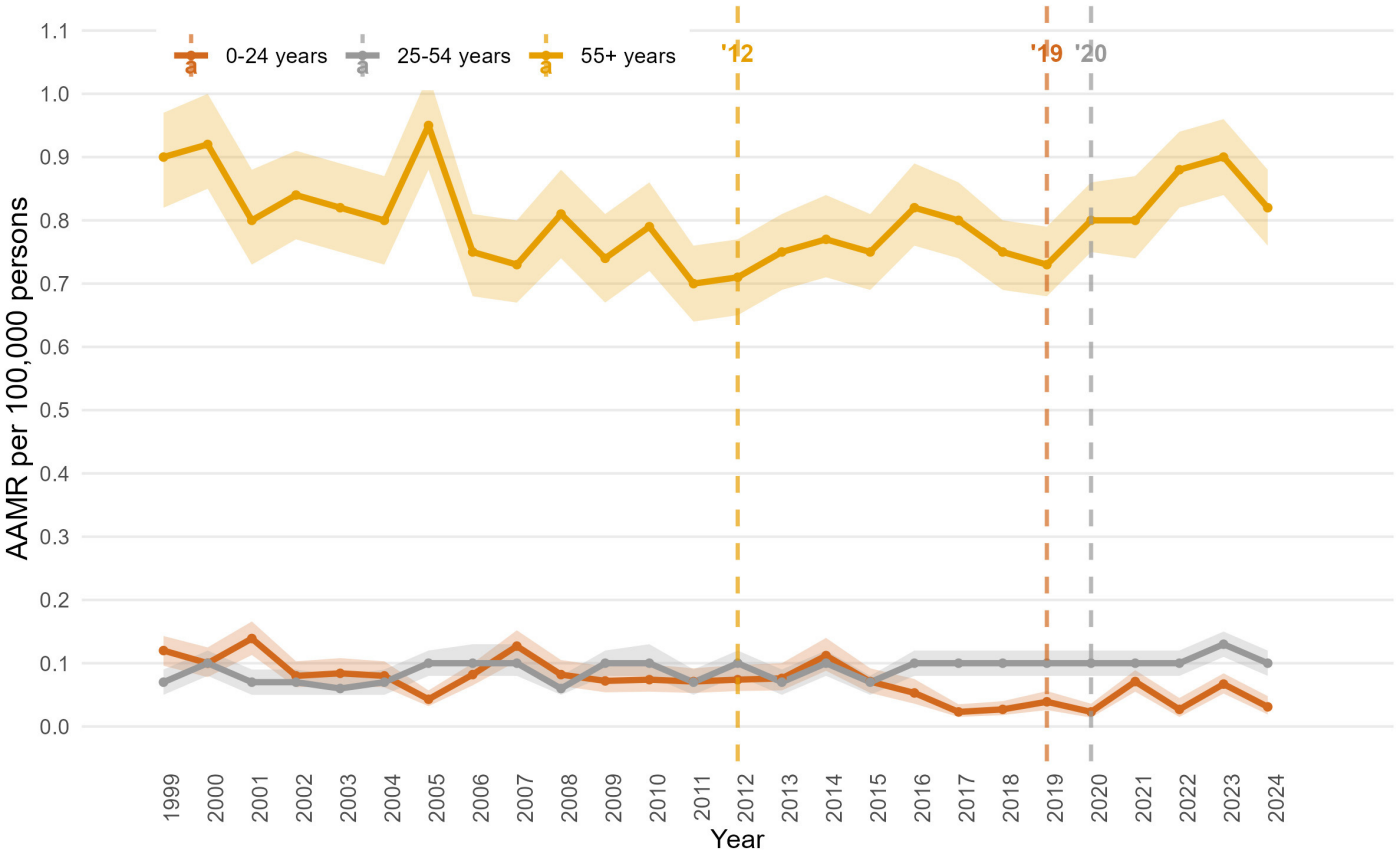
Age-stratified trends in age-adjusted mortality rates for hydrocephalus, 1999–2024 (*N* = 19650; 0–24 years old: 1472; 25–54 years old: 2532; 55+ years old: 15646).

From 1999 to 2024, the AAMR among individuals aged 0–24 years fell from 0.12 (95% CI: 0.10 – 0.14) to 0.03 (95% CI: 0.02 – 0.05), yielding an AAPC of −2.88 (95% CI: −8.96 – 3.59, *P* = 0.37). Temporal patterns for this youngest cohort exhibited two distinct segments: 1999–2019, with an APC of −4.03 (95% CI: −6.84 to −1.13, *P* = 0.009), followed by 2019–2024, where the APC was 1.81 (95% CI: −26.16 – 40.36, *P* = 0.91). For those aged 25–54 years, the AAMR rose from 0.07 (95% CI: 0.05–0.09) in 1999 to 0.10 (95% CI: 0.08 – 0.12) in 2024, corresponding to an AAPC of 1.42 (95% CI: −0.91 – 3.80, *P* = 0.235). The trend for this middle-aged group showed: 1999–2020, with an APC of 0.99 (95% CI: −0.31 – 2.31, *P* = 0.13), and 2020–2024, with an APC of 3.70 (95% CI: −9.69 – 19.07, *P* = 0.59). Among participants aged 55 years and older, the AAMR declined from 0.90 (95% CI: 0.82–0.97) to 0.82 (95% CI: 0.76–0.88) across the study horizon, yielding an AAPC of −0.15 (95% CI: −0.81 – 0.51, *P* = 0.656). The oldest age group also experienced a changing trajectory: an initial fall during 1999–2012 with an APC of −1.57 (95% CI: −2.55 –−0.58, *P* = 0.004), followed by a subsequent rise from 2012 to 2024 showing an APC of 1.41 (95% CI: 0.42 – 2.41, *P* = 0.007). These findings are illustrated in Figure 3 and comprehensively presented in Tables 2, 3.

Due to CDC's data suppression policy, counts based on fewer than 10 deaths are not reported. Therefore, the analytic sample size for subgroup analyses (*N* = 19,650) is smaller than the total number of identified deaths (*N* = 20,142). AAMR: Age-Adjusted Mortality Rates.

### 3.4 Trends by ethnicity

**Figure 4:**
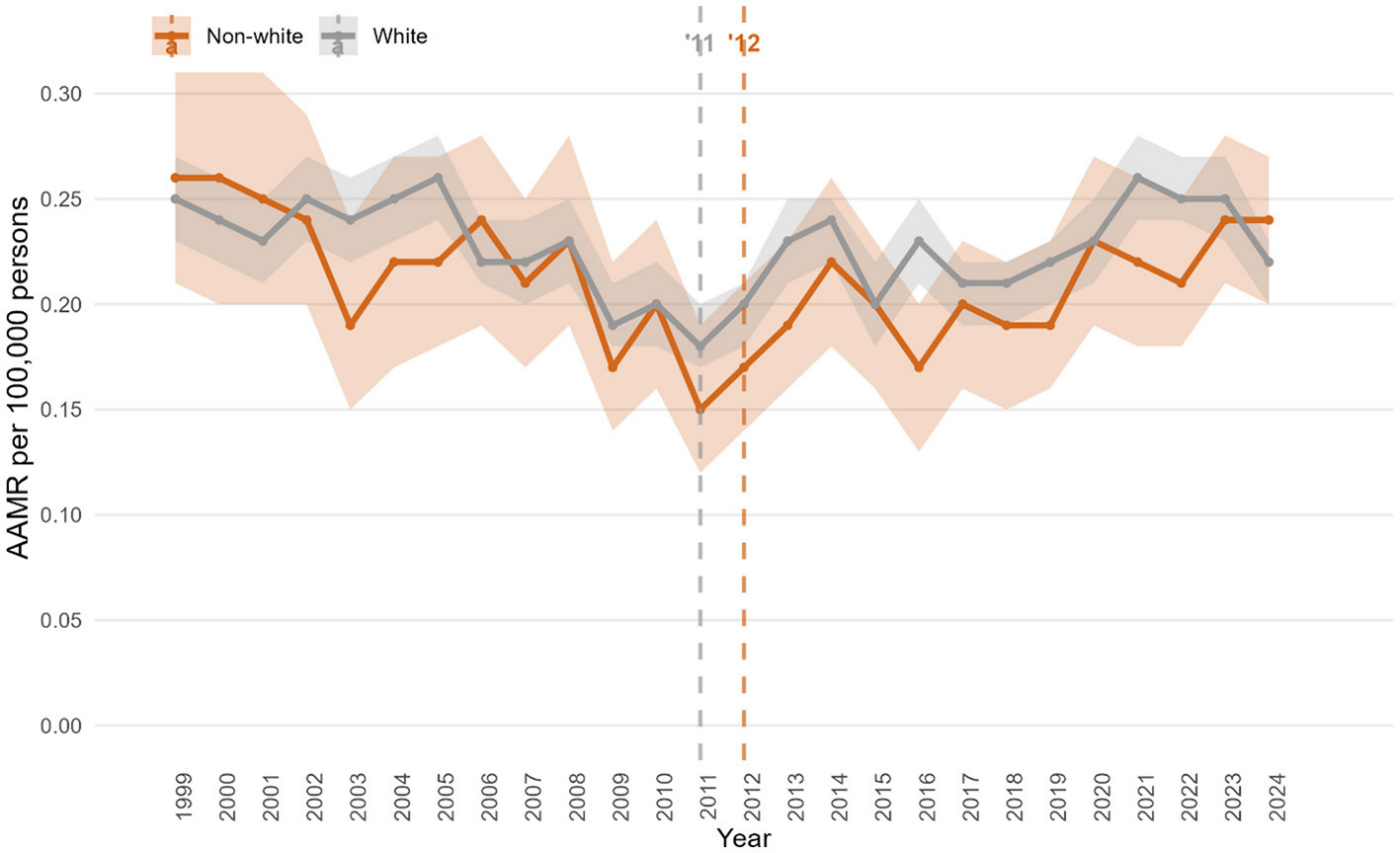
Ethnicity–stratified trends in age-adjusted mortality rates for hydrocephalus, 1999–2024 (*N* = 20,142; non-White: 3110; White: 17032). AAMR: age-adjusted mortality rates.

Between 1999 and 2024, the AAMR among non-white individuals declined from 0.26 (95% CI: 0.21 – 0.31) to 0.24 (95% CI: 0.20 – 0.27), corresponding to an AAPC of −0.40 (95% CI: −1.48 – 0.70, *P* = 0.474). This ethnic subgroup demonstrated two distinct phases: 1999–2012, with an APC of −2.68 (95% CI: −4.31 to −1.02, *P* = 0.003), followed by 2012–2024, where the APC was 2.14 (95% CI: 0.55 – 3.75, *P* = 0.01). Regarding white Americans, their AAMR likewise dropped across the study period, from 0.25 (95% CI: 0.23 – 0.27) in 1999 to 0.22 (95% CI: 0.20 – 0.23) in 2024, translating to an AAPC of −0.16 (95% CI: −0.97 – 0.66, *P* = 0.702). This ethnic subgroup also underwent a shifting trend: a reduction during 1999–2011 with an APC of −1.77 (95% CI: −3.05 to −0.48, *P* = 0.009), then an increase from 2011 to 2024 with an APC of 1.35 (95% CI: 0.21 – 2.52, *P* = 0.02). These results are displayed in Figure 4 and fully detailed in Tables 2, 3.

### 3.5 Trends by census regions

**Figure 5:**
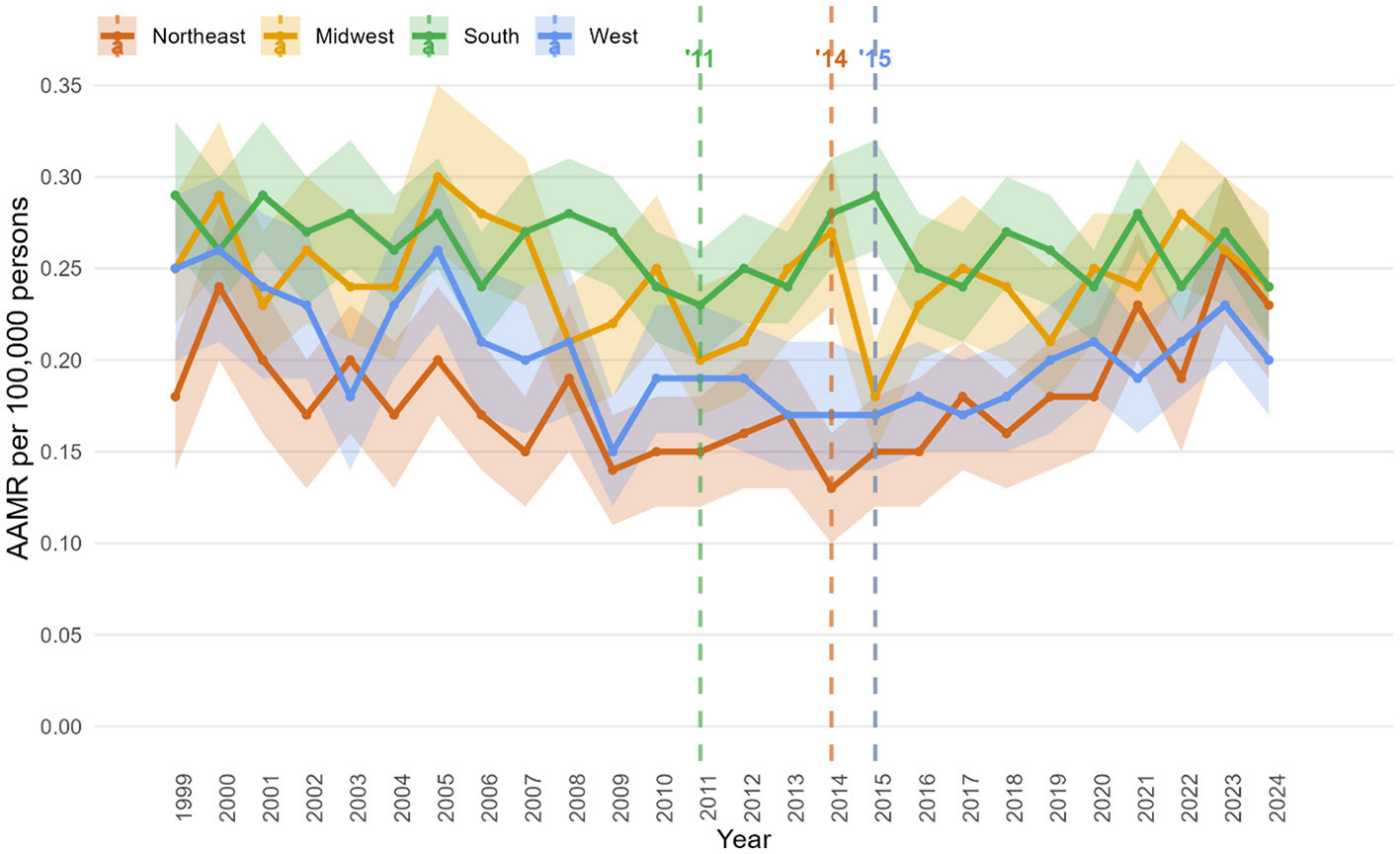
Census regions-stratified trends in age-adjusted mortality rates for hydrocephalus, 1999–2024 (*N* = 20,166; Northeast: 3133; Midwest: 4794; South: 8287; West: 3952). AAMR: age-adjusted mortality rates.

All four census regions exhibited varying trends in AAMR from 1999 to 2024. In the Northeast, the AAMR declined from 0.18 (95% CI: 0.14 – 0.21) in 1999 to 0.23 (95% CI: 0.19 – 0.26) in 2024, corresponding to an AAPC of 0.57 (95% CI: −0.67 – 1.82, *P* = 0.368). Temporal patterns for this region revealed two phases: 1999–2014, with an APC of −2.79 (95% CI: 4.17 to −1.40, *P* < 0.001), followed by 2014–2024, where the APC was 5.83 (95% CI: 3.24 – 8.49, *P* < 0.001). For the Midwest, the AAMR dropped from 0.25 (95% CI: 0.22 – 0.29) to 0.24 (95% CI: 0.21 – 0.28) across the study horizon, yielding an AAPC of −0.16 (95% CI: −1.35 – 1.04, *P* = 0.794). This region demonstrated a clear shift: 1999–2015, with an APC of −0.99 (95% CI: −2.14 – 0.18, *P* = 0.092), and 2015–2024, with an APC of 1.33 (95% CI: −1.52 – 4.27, *P* = 0.347). In the South, the AAMR fell from 0.29 (95% CI: 0.26 – 0.33) in 1999 to 0.24 (95% CI: 0.21 – 0.26) in 2024, translating to an AAPC of−0.38 (95% CI: −1.16 – 0.40, *P* = 0.334). The Southern trajectory showed: 1999–2011, with an APC of 1.26 (95% CI: −2.62 – 0.12, *P* = 0.071), and 2011–2024, with an APC of 0.43 (95% CI: −0.51 – 1.39, *P* = 0.354). For the West, the AAMR decreased from 0.25 (95% CI: 0.20 – 0.29) in 1999 to 0.20 (95% CI: 0.17 – 0.23) in 2024, corresponding to an AAPC of −0.58 (95% CI: −1.49 – 0.34, *P* = 0.216). This region also experienced a two-stage pattern: an initial decline during 1999–2015 with an APC of −2.55 (95% CI: −3.56 to −1.52, *P* < 0.001), followed by a subsequent rise from 2015 to 2024 showing an APC of 3.01 (95% CI: 1.00 – 5.07, *P* = 0.005). Geographic disparities are presented in Figure 5 and fully described in Tables 2, 3.

### 3.6 Mortality trends before and during the COVID-19 pandemic

To quantify the impact of the COVID-19 pandemic on hydrocephalus-related mortality, we compared AAMR trends between the pre-pandemic period (1999–2019) and the pandemic period (2020–2023) by fixing a joinpoint at 2019. Overall, the AAMR showed a significant decline during 1999–2019 (AAPC = −0.87, 95% CI: −1.40 to −0.33, *P* = 0.003), followed by a reversal to an increasing trend during 2020–2023 (AAPC = 3.61, 95% CI: −0.97 to 8.41, *P* = 0.12), although the latter did not reach statistical significance. By sex, both females and males experienced significant increases during the pandemic period (females: AAPC = 1.64, 95% CI: 0.43–2.86, *P* = 0.009; males: AAPC = 1.15, 95% CI: 0.08–2.24, *P* = 0.04), following non-significant declines in the pre-pandemic period. By age group, the most striking reversal was observed among adults aged 55+ years, whose AAMR shifted from a non-significant decline before the pandemic (AAPC = −0.54, 95% CI: −1.23–0.16, *P* = 0.13) to a significant increase during the pandemic (AAPC = 1.41, 95% CI: 0.42–2.41, *P* = 0.007). In contrast, the youngest age group (0–24 years) and adults aged 25–54 years did not show statistically significant changes during either period, likely due to small case numbers and wide confidence intervals. By ethnicity, non-White populations showed a significant increase during the pandemic (AAPC = 2.14, 95% CI: 0.55–3.75, *P* = 0.01), whereas White populations also experienced a significant increase (AAPC = 1.35, 95% CI: 0.21–2.52, *P* = 0.02). Both racial groups had non-significant declines before the pandemic. Geographically, the Northeast region experienced the largest increase during the pandemic (AAPC = 5.83, 95% CI: 3.24–8.49, *P* < 0.001), followed by the West (AAPC = 3.01, 95% CI: 0.10–5.07, *P* = 0.005). The Midwest and South regions showed non-significant increases during the pandemic (Midwest: AAPC = 1.33, *P* = 0.35; South: AAPC = 0.43, *P* = 0.35), with stable trends in the pre-pandemic period. Collectively, these findings suggest that the COVID-19 pandemic may have interrupted the previously declining or stable mortality trajectory for hydrocephalus, with differential impacts across demographic and geographic subgroups. The reversal was most pronounced among older adults, females, non-White populations, and residents of the Northeast and West regions (Table 4).

**Table 4:** Mortality trends for hydrocephalus before and during the COVID-19 pandemic.

Characteristic	Segment 1			Segment 2		
	Year	APC (95% CI)	*P*-Value	Year	APC (95% CI)	*P*-Value
Overall	1999–2019	−0.87 (−1.40, −0.33)	0.003	2020–2023	3.61 (−0.97, 8.41)	0.12
Sex						
Female	1999–2019	−0.96 (−2.07, 0.13)	0.08	2020–2023	1.64 (0.43, 2.86)	0.009
Male	1999–2019	−0.99 (−1.91, −0.07)	0.04	2020–2023	1.15 (0.08, 2.24)	0.04
Age						
0–24 years	1999–2019	−4.03 (−6.84, −1.13)	0.009	2020–2023	1.81(−26.16, 40.36)	0.91
25–54 years	1999–2019	0.99 (−0.31, 2.31)	0.13	2020–2023	3.70(−9.69, 19.07)	0.59
55+ years	1999–2019	−0.54 (−1.23, 0.16)	0.13	2020–2023	1.41(0.42, 2.41)	0.007
Race						
Non-white	1999–2019	−1.02 (−2.16, 0.13)	0.08	2020–2023	2.14 (0.55, 3.75)	0.01
White	1999–2019	−0.53 (−1.38, 0.32)	0.22	2020–2023	1.35(0.21, 2.52)	0.02
Census regions						
Midwest	1999–2019	−0.53 (−1.55, 0.50)	0.31	2020–2023	1.33 (1.52, 4.27)	0.35
Northeast	1999–2019	−0.71 (−1.86, 0.46)	0.23	2020–2023	5.83 (3.24, 8.49)	<0.001
South	1999–2019	−0.59 (−1.44, 0.27)	0.18	2020–2023	0.43 (−0.51, 1.39)	0.35
West	1999–2019	−1.46 (−2.32, −0.59)	<0.001	2020–2023	3.01 (0.10, 5.07)	0.005

### 3.7 Distribution of state-level mortality

**Figure 6:**
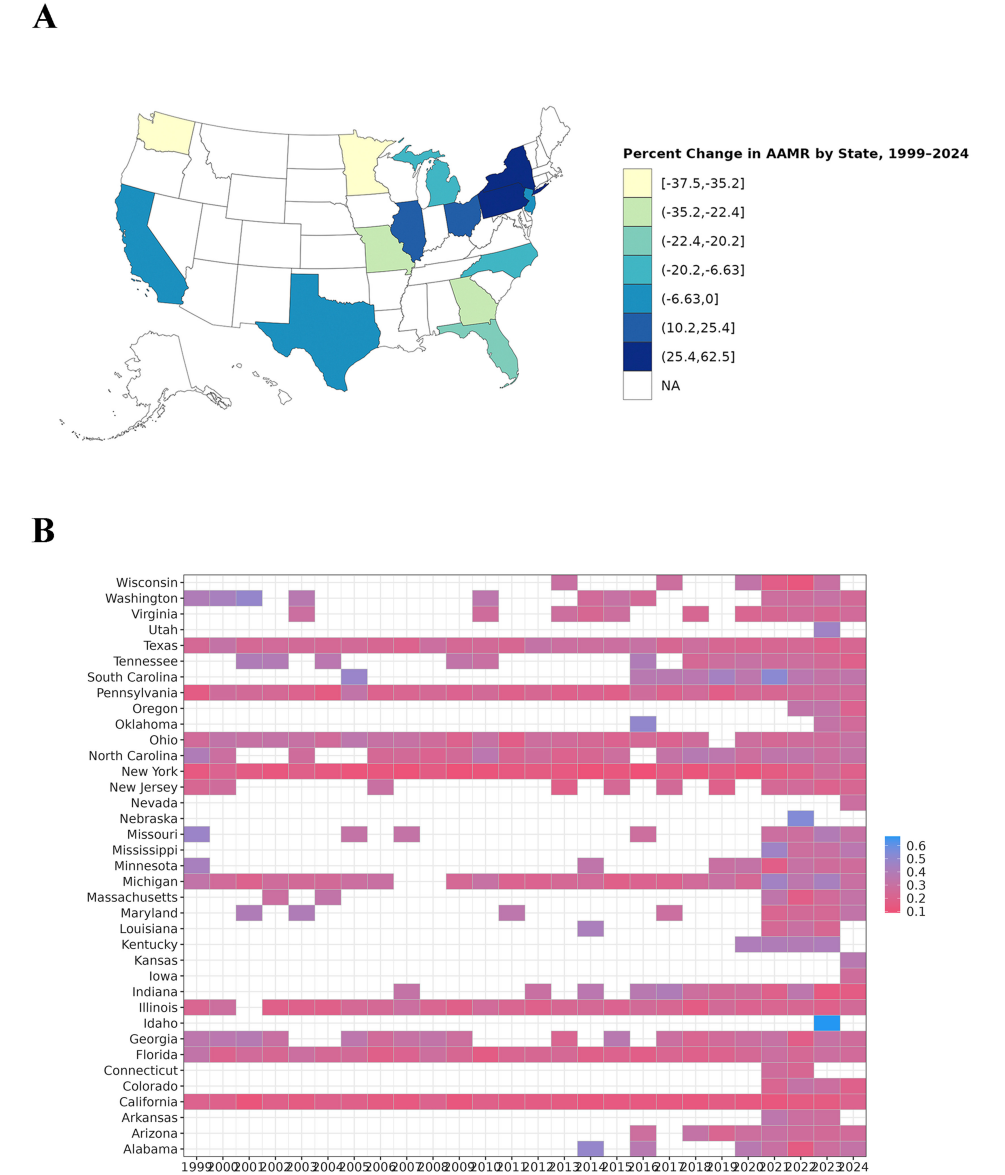
Geographic distribution and time trends in death rates across American States (*N* = 13,000). **(A)** Choropleth map showing the geographic distribution of percentage changes in AAMR for hydrocephalus by state from 1999 to 2024. States in gray (NA) indicate data were suppressed due to small case numbers (< 20 deaths); consequently, the percentage change intervals are not continuous. **(B)** Heatmap of state-specific AAMR for hydrocephalus from 1999 to 2024, with each row representing a state and color intensity reflecting the mortality rate in that state for a given year. State-level analyses were based on deaths with non-suppressed counts. Due to CDC WONDER's data suppression policy for counts < 10, the analytic sample for state-level analyses was 13,000 deaths, which is smaller than the total of 20,142 deaths identified nationally.

In the spatial analysis, we mapped the percentage change in deaths across U.S states by SPLS (A statistical platform for life sciences) (21) from 1999 to 2024 (Figure 6A). Due to relatively few deaths in certain states, state-level percentage changes are subject to statistical instability and should be interpreted as approximate trends rather than precise measures. Oklahoma and West Virginia suggested the steepest increases, with changes falling within the highest interval of 25.4% to 62.5%. Texas, Florida, and California showed moderate growth, corresponding to the 10.2% to 25.4% range. In contrast, several states in the Northeast, including New York and Massachusetts, showed estimated declines in the −35.2% to −22.4% bracket. A handful of states, such as Iowa and Nebraska, suggested relatively stable trends with changes between −6.63% and 0%. States with unreliable estimates due to small case numbers were labeled as NA. The geographic distribution of AAMR across states by SPLS from 1999 to 2024 is illustrated in Figure 6B.

## 4 Discussion

Between 1999 and 2024, the trajectory of AAMR across all examined sex, age, ethnicity, and region subgroups was characterized by a consistent biphasic pattern: an early decline followed by a recent increase. This pattern was consistently observed across most subgroups, although the timing of the turning point varied—occurring around 2009–2015 depending on the subgroup. Notably, declines during the early period were statistically significant for females (1999–2009: APC = −3.52, *P* = 0.001), males (1999–2012: APC = −2.13, *P* = 0.004), adults aged ≥55 years (1999–2012: APC = −1.57, *P* = 0.004), both racial groups, and the Northeast and West regions. Subsequent increases were also statistically significant among these subgroups during their respective later periods (e.g., Northeast 2014–2024: APC = 5.83, *P* < 0.001; non-White 2012–2024: APC = 2.14, *P* = 0.01). While the overall 26-year AAPC did not reach statistical significance (0.01, *P* = 0.98), this overall stability reflected the averaging of these opposing phases rather than an absence of meaningful change. The consistency of this reversal pattern across diverse subgroups—observed both before and during the COVID-19 pandemic—suggests a broader shift in the mortality burden of hydrocephalus that warrants further investigation.

Our research found that AAMR was consistently greater among male relative to female. Female has been identified as an independent risk factor for the development of hydrocephalus after aneurysmal subarachnoid hemorrhage (22, 23). However, current evidence does not support a significant difference in hydrocephalus-related mortality between sexes. A multicenter study in the U.S. demonstrated that male hydrocephalus patients had a higher likelihood of receiving treatment, whereas no significant sex disparities were identified in post-treatment outcomes (24). The ratio of males to females showed no significant change over time, reflecting a persistent disparity. After 2020, the number of hydrocephalus-related deaths increased substantially in both males and females. However, our analysis revealed a continuous increase in mortality counts for both sexes over the past decade. Therefore, the marked rise observed after 2020 may not necessarily be causally linked to the COVID-19 pandemic, as causal inference cannot be drawn from current data.

According to our study, White individuals had higher AAMR than non-White populations in the majority of years, although non-White rates exceeded White rates in certain years (1999, 2000, 2001, 2006, and 2024). Both ethnic groups exhibited a pattern of early decline followed by recent increase, with non-White populations showing steeper changes in both directions. Interestingly, a study revealed that, after adjusting for confounding factors, mortality rates were higher among Hispanic, Black, and other non-White children with hydrocephalus, whereas Hispanic children received fewer shunt revision procedures (25). Moreover, among preterm infants with posthemorrhagic hydrocephalus, Black infants have a significantly higher mortality rate than their White and Hispanic counterparts (26). Additionally, a body of evidence derived from large database analyses indicates that Black children with hydrocephalus experience higher death risks compared to White children. Specifically, this population shows increased mortality from both congenital and acquired hydrocephalus, elevated fatality associated with post-hemorrhagic hydrocephalus of prematurity, and a greater chance of non-routine hospital departure (27, 28). These observations highlight important and potentially underrecognized disparities in the management of hydrocephalus in the pediatric population. The aforementioned studies indicate that non-White populations experience higher mortality, which appears to contradict the trend observed in our study. Of note, those studies focused primarily on children. Given that mortality rates may be influenced by socioeconomic and other factors, further analysis is warranted to clarify the role of ethnicity in these disparities.

Our analysis revealed that the AAMR for hydrocephalus patients aged 55+ years and older was significantly higher than that for the two younger groups (0–24 and 25–54 years), with no significant difference in mortality observed between the latter two. This finding suggests that advanced age may be a significant factor influencing hydrocephalus-related mortality. A clear age-related analysis in hydrocephalus prevalence was observed. The older adults population had the highest prevalence (175/100,000), followed by the pediatric group (88/100,000), whereas adults had the lowest prevalence (11/100,000). This suggests that age is a significant determinant of hydrocephalus burden (11). One study found that advanced age (≥60 years) increases the risk of poor outcomes in patients with aneurysmal subarachnoid hemorrhage only when hydrocephalus is also present. In the absence of hydrocephalus, older age alone does not predict a worse prognosis (29). The average age of patients admitted to the intensive care unit (ICU) has shown an upward trend over time. Despite the absence of a causal link between age and clinical outcomes, age continues to serve as a commonly employed predictor of unfavorable outcomes in the ICU (30). A study on hospitalization rates for normal pressure hydrocephalus showed that older patients had higher rates of hospitalization (31). This finding may support our conclusion that advanced age is linked to increased mortality. Of note, the rise in mortality after 2020 was most evident in the population aged 55+ years, potentially suggesting a link to the COVID-19 pandemic.

From a geographic perspective, hydrocephalus-related mortality across U.S. regions (Northeast, Midwest, South, and West) did not exhibit persistent or stable regional differences during the study period. Although certain regions showed slightly higher or lower values in some years, overall, the rates fluctuated across regions without a consistent geographic gradient or a persistently dominant region. Although no study to date has demonstrated regional differences in hydrocephalus-related mortality across the United States, a global systematic review and meta-analysis revealed significant geographic disparities in hydrocephalus epidemiology. The incidence of congenital hydrocephalus was highest in Africa (145 per 100,000 births) and Latin America (316 per 100,000 births), and lowest in the United States/Canada (68 per 100,000 births). Moreover, low- and middle-income countries had a higher incidence (123 per 100,000 births) compared to high-income countries (79 per 100,000 births). The greatest burden falls on Africa, Latin America, and Southeast Asia (10). This finding suggests that geographic location alone may not be a major determinant of hydrocephalus-related mortality. Several factors may explain this observation. First, the diagnosis, treatment, and long-term management of hydrocephalus rely heavily on access to and quality of healthcare resources. Although regional differences in these resources exist across the United States, they may not be substantial enough to consistently impact mortality rates. Second, patients with hydrocephalus often require referral to specialized centers, and cross-regional healthcare-seeking behavior may mitigate the influence of geographic location. Finally, with continuous advances in neurosurgical techniques and perioperative care, hydrocephalus-related mortality has remained relatively low, which may dilute any potential geographic disparities.

The COVID-19 pandemic may have contributed to the observed reversal in hydrocephalus mortality trends, although the underlying mechanisms are likely indirect. Unlike acute infectious diseases, hydrocephalus itself is not directly affected by SARS-CoV-2 infection; rather, the pandemic may have disrupted routine healthcare delivery for this chronic condition. Elective neurosurgical procedures, including ventriculoperitoneal shunt placements and revisions, were frequently postponed or canceled during the early pandemic period due to hospital capacity constraints and infection control measures. Delayed surgical interventions may have led to uncontrolled intracranial pressure and subsequent complications, ultimately increasing mortality risk among hydrocephalus patients. The differential impact across demographic subgroups warrants further attention. The significant increase among older adults (aged 55+ years) during the pandemic may reflect their greater vulnerability to healthcare disruptions, as this population often requires more frequent neurosurgical follow-up and has a higher prevalence of comorbidities that increase the risk of severe outcomes when care is delayed. The rising trends observed among both racial groups, particularly non-White populations, may also point to pre-existing disparities in healthcare access that were exacerbated during the pandemic. Geographic heterogeneity was also evident, with the Northeast and West regions experiencing the largest increases during the pandemic. These regions were among the earliest and most severely affected by COVID-19 outbreaks in the United States, which may have led to more substantial healthcare system strain and subsequent disruptions to neurosurgical services. Alternatively, these regions may have more complete reporting of hydrocephalus-related deaths, contributing to the observed increases. Importantly, the pandemic-related increase in AAMR observed during 2020–2023 did not reach statistical significance at the national level, suggesting that the overall mortality burden of hydrocephalus may have been relatively resilient to the pandemic shock. However, the consistent reversal in trend direction—from a significant decline before the pandemic to an increase during the pandemic—across multiple subgroups is clinically noteworthy and warrants continued surveillance to determine whether these trends persist or reverse as healthcare systems stabilize. While several case reports have documented the occurrence of hydrocephalus or hydrocephalus-related complications in patients with acute COVID-19 infection (32–34), systematic evidence on whether COVID-19 directly contributed to mortality among hydrocephalus patients remains limited. A study examining patients with shunted hydrocephalus found that COVID-19 infection was associated with an almost five-fold increase in shunt revision rates and significantly higher mortality, suggesting that COVID-19 may exacerbate clinical deterioration in this vulnerable population (35). However, large-scale population-based studies examining the pandemic's impact on hydrocephalus mortality trends are still scarce. Our study provides, to our knowledge, one of the first population-level assessments demonstrating that the COVID-19 pandemic period (2020–2023) was associated with a reversal of the previously declining mortality trajectory for hydrocephalus. These findings add to the growing body of evidence that COVID-19 may have indirect but meaningful effects on hydrocephalus outcomes, likely through healthcare disruptions and increased clinical vulnerability, rather than through direct viral pathogenicity.

### 4.1 Limitations

This investigation has several limitations. First, this analysis is ecological in nature and relies on aggregated mortality or incidence data; therefore, causal relationships cannot be inferred at the individual level. Second, disease burden estimates depend on registry data or hospital discharge records, which may carry a risk of misclassification, and diagnostic criteria or coding practices may differ across regions or time periods. Third, the CDC WONDER database does not contain patient-level clinical details, including etiologic subtypes of hydrocephalus (e.g., congenital, acquired, or normal pressure hydrocephalus), prior surgical history, presence of major comorbidities (such as diabetes, hypertension, or cardiovascular disease), or duration of intensive care unit stay—all of which are known to significantly influence mortality outcomes. The absence of these clinical data, along with socioeconomic and behavioral information, limits our ability to adjust for key confounders and may have introduced bias into our mortality estimates, hindering in-depth mechanistic interpretation of the observed disparities. Fourth, although joinpoint regression can detect statistically significant turning points in temporal trends, it does not clarify the underlying factors driving those changes. Fifth, the available data only extend through a specific year (e.g., 2024); thus, more recent patterns require ongoing surveillance and updated analyses. Furthermore, because hydrocephalus encompasses heterogeneous subtypes with diverse etiologies (e.g., communicating, obstructive, and normal pressure hydrocephalus), and because the underlying pathophysiology varies considerably across age groups, the overall trends observed may reflect differential contributions from each subtype. Our study was not designed to explore subtype-specific mechanisms or to attribute changes to any particular pathophysiologic process. Despite these limitations, the use of standardized methodology applied to long-term, nationally or regionally representative data provides robust and valuable insights into the long-term epidemiologic patterns of hydrocephalus.

## 5 Conclusion

Between 1999 and 2024, the AAMR trajectories across sex, age, ethnicity, and region subgroups revealed a comparable reversal pattern: an initial decrease followed by a subsequent rise. This trend was evident across most subgroups, with the most notable turnarounds occurring among older adults (≥55 years), non-White populations, and residents of the Northeast and West regions. Although the overall 26-year AAPC was not statistically significant (0.01, *P* = 0.98), this overall stability masked a consistent pattern of early decline followed by recent increase across most subgroups, rather than an absence of meaningful change. Whether this reversal reflects COVID-19-related healthcare disruptions, changes in disease burden, or other unmeasured factors remains unclear and merits further exploration. These findings highlight the need for continued surveillance of hydrocephalus mortality trends. From a policy perspective, they may also point to the value of maintaining access to specialized neurosurgical care, particularly for vulnerable populations. However, further research would be needed to determine whether targeted structural interventions could effectively reduce the observed mortality disparities across subgroups.

## Data Availability

The original contributions presented in the study are included in the article/supplementary material, further inquiries can be directed to the corresponding author.
